# A comparison of supraglottic airway devices versus endotracheal intubation for use in rabbit anaesthesia

**DOI:** 10.18849/ve.v7i3.563

**Published:** 2022-07-06

**Authors:** Sarah Daphne Foo+

**Keywords:** AIRWAY MAINTENANCE, ANAESTHESIA, COMPARISON, INTUBATION, LARYNGEAL MASK, RABBIT, SUPRAGLOTTIC DEVICE, V-GEL, VENTILATION

## Abstract

PICO question

In domestic rabbits undergoing anaesthesia, how does the use of supraglottic airway devices compare to endotracheal intubation for ease of use in achieving a patent airway and maintaining a stable anaesthesia?

Clinical bottom line

Category of research question

Treatment

The number and type of study designs reviewed

Five papers were reviewed to answer this clinical question including four randomised controlled trials, one of which was a randomised crossover trial and one peer-reviewed conference proceeding

Strength of evidence

Moderate

Outcomes reported

There is evidence to support that supraglottic devices were easier and faster to insert than endotracheal tubes and were used effectively to achieve and maintain a patent airway and anaesthesia. They were however, more easily displaced and took up more space in the oral cavity. Evidence also supports endotracheal intubation can be used to effectively achieve a patent airway and maintain a stable anaesthesia however, can result in more damage to tracheal mucosa when attempted blindly and required higher doses of induction drugs to use

Conclusion

Based on current available evidence, endotracheal intubation is an excellent option for maintaining a patent airway and anaesthesia in rabbit patients as it is a tried and tested method, however, can cause tracheal damage if conducted blindly. Supraglottic airways devices can be used as an alternative where endotracheal intubation is unsuccessful. They can also be used where speed of obtaining a patent airway is imperative such as in an emergency as they may be easier and faster to apply, especially in inexperienced practitioners without the necessary equipment for safe endotracheal intubation. Supraglottic devices are unsuitable for procedures that require access to the oral cavity and / or patient movement, due to the size and easier loss of seal during movement potentiating risk of aspiration. Both supraglottic devices and endotracheal intubation are superior to face masks which evidence shows have more leakage, dead space and increased risk of hypercapnia

How to apply this evidence in practice

The application of evidence into practice should take into account multiple factors, not limited to: individual clinical expertise, patient’s circumstances and owners’ values, country, location or clinic where you work, the individual case in front of you, the availability of therapies and resources.

Knowledge Summaries are a resource to help reinforce or inform decision making. They do not override the responsibility or judgement of the practitioner to do what is best for the animal in their care.

## Clinical scenario

Rabbits are becoming more popular as pets and as their numbers increase, so does the requirement for veterinary care including procedures that require a general anaesthetic. There is a relatively high anaesthetic death risk for rabbits of up to eight times greater of that of cats and dogs (Brodbelt et al., 2008). Complications one may experience with endotracheal intubation in rabbits include airway obstruction, damage to the tracheal mucosa, oesophageal perforation and in some cases death as a result of tracheitis or airway obstruction (Phaneuf et al., 2006; Grint et al., 2006; and Ranchère et al., 1992). Endotracheal intubation can be complex in rabbits due to the restricted size of the oropharynx, the position of the tongue and laryngeal spasms which can be exacerbated when using a blind technique instead of visualising the trachea using a modified otoscope, laryngoscope, or endoscope (Manning et al., 1994; Benito et al., 2021; Tran et al., 2001; Thompson et al., 2017; and Corleta et al., 1992). Supraglottic devices such as laryngeal mask airway devices are commonly used in human medicine, and prevalence of use is increasing in animal medicine (Cook, 2003; and Crotaz, 2010). The rabbit specific supraglottic airway device (SGAD), the v-gel®, was developed as an alternative to laryngeal mask airway devices to try to reduce complications such as lingual cyanosis (Kazakos et al., 2007). The v-gel® has been demonstrated to be suitable to maintain a patent airway in rabbit anaesthesia and may be faster to insert than endotracheal tubes (ETT) (Bateman et al., 2005; and Crotaz, 2013). This is especially useful in a clinical setting where achieving a patent airway quickly is imperative such as in respiratory arrest, or where multiple endotracheal intubation attempts have been unsuccessful. This Knowledge Summary investigates the differences between endotracheal intubation and placement of a supraglottic airway device when maintaining anaesthesia and a patent airway in rabbit patients.

## The evidence

The studies reviewed included four randomised controlled trials, one of which was a randomised crossover trial and one peer-reviewed conference proceeding. Randomised controlled trials are strong study designs however all studies had small sample sizes which would have affected the power of the results. The studies looked at only two breeds, Norfolk rabbits and New Zealand White rabbits, making it difficult to generalise the results to the general rabbit population (Cruz et al., 2000; Engbers et al., 2017; Comolli et al., 2020; Toman et al., 2015; and Wenger et al., 2017). All studies also only looked at healthy, adult rabbits with low anaesthetic risk based on the American Society of Anesthesiologists (ASA) (2020) physical status grading scale. Only one study assessed the difference between endotracheal intubation and use of supraglottic airway devices whilst the subjects were undergoing a surgical procedure common in general practice. Thus, the results of the other studies may be less representative of in clinic rabbit patients undergoing anaesthesia for surgery. The main outcomes considered between the devices were mucosal damage, loss of airway seal, ability to achieve positive pressure ventilation and ease of application. A consistent finding across all studies is that the application of a supraglottic airway device was faster than intubation with an endotracheal tube, however this may be due to lack of training. All studies demonstrated that anaesthetic parameters such as heart rate, respiratory rate, temperature and depth did not differ depending on which device was used. The studies also showed that supraglottic devices were more easily displaced during patient movement and that endotracheal intubation could cause more mucosal damage if using the blind technique. Both airway devices have evidence supporting their effective use in rabbit anaesthesia and taking into account the limitations and benefits of each device will allow the general practitioner to choose the best option for their patients.

## Summary of the evidence

**Engbers et al. (2017):** 

Population:	Adult New Zealand White laboratory rabbits with no indication of systemic illness (ASA classification status of 2 or less).
Sample size:	Initial sample size: 15 rabbits. Final sample size: 13 rabbits (reasons for exclusion addressed in ‘Intervention details’).
Intervention details:	Subjects were acclimated to environment for 1 week prior to the experiment. The subjects were allocated to groups via randomised block selection: v-gel® device (supraglottic airway device [SGAD]) – seven rabbits. Orotracheal intubation (endotracheal tube [ETT]) – six rabbits. Subjects were fasted for 2 hours and underwent a physical exam prior to the experiment. All subjects were premedicated with a mixture of midazolam and dexmedetomidine intramuscularly with alfaxalone used for induction. Two CT scans were taken, one baseline before insertion, and one 10 minutes after the devices were inserted. The scans were from the margin of the nose to the thoracic inlet. The SGAD devices were selected based on the subject mass. Lubricating spray was applied to the larynx prior to insertion with the tongue positioned outside the mouth. The device was inserted until resistance was felt or when the device fixation tabs were positioned within 1–2cm of the incisors. A capnograph was used to confirm successful placement. The ETT group was intubated with endotracheal tubes using the blind technique with sizes being selected based on the experience of the operator. The subjects were placed in sternal recumbency with hyperextension of the head and neck. Lidocaine was sprayed into the oropharynx 30 seconds prior to intubation. Placement was confirmed with a positive capnograph reading. Both SGAD and ETT devices were secured behind the ears with a bandage tie. Blood pressure, heart rate, respiratory rate and oxygen saturation were recorded every 30 seconds for 5 minutes and at 15 minutes after the devices were inserted. At 15 minutes isoflurane concentration was measured 5 cm away from the mouth. The airway seal was tested by closing the airway valve and squeezing the reservoir bag to assess for leakage. In four SGAD and five ETT subjects, arterial blood samples were collected to measure blood gasses and electrolytes. The subjects were maintained under general anaesthesia for a total of 60 minutes using a Bain non-rebreathing system. All subjects were euthanised at the conclusion of the anaesthetic with intravenous sodium pentobarbital. One operator was used for each group. Exclusions: Two rabbits were excluded from the results, one due to failure of intubation and the other due to prolonged intubation time due to presence of faecal matter in the oropharynx.
Study design:	Randomised controlled trial.
Outcome studied:	Time to successful device insertion: Measured from when the SGAD or ETT passed the incisors to confirmation of six waveforms using a capnograph. Number of attempted device placements: In the absence of a positive capnograph signal, adjustments were made, and the number of attempts was recorded. Narrowest region of upper airway: In the SGAD group, Computed Tomography (CT) scans were used to assess the narrowest region of the upper airway in relation to the tip of the SGAD. In the ETT group, CT scans were used to measure the cross-sectional area of the lumen. Airway sealing pressure: Assessed by closing the circuit and squeezing the reservoir bag with peak inspiratory pressures being increased by 5 cmH_2_O increments until reaching 20 cmH_2_ Histological score of tracheal tissue: Necropsies were performed on all subjects up to 2 hours following euthanasia by a pathologist (blinded). The tongue, trachea, pharynx and larynx were fixed in formalin and stained with haematoxylin and eosin (H&E). The following areas were examined: Larynx at the level of vocal folds. Trachea immediately caudal to larynx. Trachea 1 cm caudal to larynx. A histological score of tracheal tissue was given with scores for the sections from the same regions (above) averaged to give a total score out of six: 0 = Normal. 1 = Mild focal erosion of mucosa with little or no leukocytic infiltration and minimal to mild locally extensive congestion of submucosa. 2 = Multifocal erosion or ulceration with oedema of lamina propria, moderate mixed leukocytic infiltration, and haemorrhage of mucosa and moderate diffuse congestion and mild perivascular oedema of submucosa. 3 = Extensive erosion or ulceration with marked mixed leukocytic infiltration, cellular debris, haemorrhage, and possibly surface exudate of the mucosa and moderate diffuse congestion and oedema with or without haemorrhage, and leukocyte infiltration associated with overlying ulcer or erosion. Arterial blood gases and electrolytes: The following were assessed: potential hydrogen (pH), partial pressure of oxygen (PaO_2_), partial pressure of carbon dioxide (PaCO_2_), base excess (BE), bicarbonate (HCO_3_), arterial oxygen saturation (SaO_2_), sodium (Na+), potassium (K+), ionised calcium, haematocrit, haemoglobin. Statistically significant differences were determined as those with a P value of less than 0.05.
Main Findings (relevant to PICO question)	Time to successful device insertion: The device insertion time was significantly shorter and more consistent in the SGAD group compared to the ETT group (P = 0.02), where SGAD device insertion had a mean of 33 seconds with a range from 14–38 seconds and ETT device placement had a mean of 59 seconds with a wider range from 29–171 seconds. Number of attempted device placements: No significant difference (P = 0.99). Narrowest region of upper airway: There was no significant distance between the caudal edge of the basihyoid bone and the rostral edge of the thyroid cartilage (P = 0.16). The cross-sectional area of the narrowest point decreased significantly between the baseline CT scan of both groups, but there was no significant difference between the ETT and SGAD groups (P = 0.93). Airway sealing pressure: The median airway seal was higher in the ETT group but there was no statistical difference (P = 0.06). Histological score of tracheal tissue: The ETT group had significantly higher mean histological score than the SGAD group where the ETT group had a mean score of 3.3 with a range of 1.0–5.0, and the SGAD group had a mean score of 0.67 with a range of 0.33–3.67 (P = 0.03). Arterial blood gases and electrolytes: There was no significant difference in any of the measured variables with hypercapnia being present in both groups (P >0.05).
Limitations:	The method of endotracheal intubation was blind. Other methods may result in reduced tracheal injury due to visualisation of the airway. The subjects were all laboratory animals of the same breed and size with a low ASA status and no surgical procedure was performed limiting the result’s extrapolation to the rabbits seen in clinical practice undergoing surgical procedures. Immediate assessment of damage to tracheal mucosa does not indicate if there are long-term effects and all subjects were euthanised following the study. This also meant assessment of recovery was not assessed which is an important factor when considering client owned rabbits. Intermittent positive pressure ventilation (IPPV) was not used in this study and thus airway sealing pressures were not assessed. Operators were trained to use the SGAD via an online training course and on two euthanised rabbits. More extensive training could have altered the results as speed of placement would likely have decreased. Operators were also the same for each group which means their technique may have improved over the course of the study, and that a direct comparison of one operator’s experience with the two different devices is not possible. Majority of outcomes showed no significant statistical difference, and this could be due to small sample size reducing power. The original sample size of 15 meant uneven randomised allocation, with the final subjects (13) also being uneven which would again affect the statistical results.

**Comolli et al. (2020):** 

Population:	Adult female New Zealand White rabbits undergoing ovariohysterectomy with American Society of Anesthesiologists (ASA) status less than 1 (excluded if any signs of illness resulting in higher ASA status). Healthy on physical examination.
Sample size:	14 rabbits.
Intervention details:	The subjects were block randomised into two groups with seven animals each: Endotracheal intubation (EEI). Rabbit specific supraglottic device, v-gel® (SGAD). The subjects were induced with ketamine, meloxicam and xylazine and maintained with isoflurane in 100% oxygen. An endoscope was used to visualise the larynx and glottis. Endotracheal tubes were lubricated and after intubation connected to capnograph with the cuff inflated until a seal was created. The v-gel® was introduced with capnograph attached until mild resistance was detected. The ovariohysterectomy was performed using standard techniques. Following anaesthetic an endoscope was used again to assess the appearance of the larynx and glottis. 4 days following study, all animals were euthanised and post-mortem examinations were performed with samples taken from the proximal and distal larynx and the tracheobronchial bifurcation. The samples were preserved in formalin, embedded in paraffin and stained with haematoxylin and eosin (H&E). Heart rate, electrocardiogram (ECG), oxygen saturation (SpO_2_), blood pressure, respiratory rate, end tidal carbon dioxide (ETCO_2_) and rectal temperature were recorded every 5 minutes. The circuit used was a modified Jackson-Rees non-rebreathing circuit, oxygen flow rate of 1–2.5 L/m.
Study design:	Randomised controlled trial.
Outcome studied:	Placement time of devices: v-gel® placement considered successful when normal waveform was present on capnograph and breathing bag showed normal movement. Endotracheal intubation considered successful when seal was formed, and positive capnograph signal seen. Number of attempts taken to achieve placement of each device. Required level of isoflurane concentrations to maintain surgical plane of anaesthesia. Arterial blood gas values: Potential hydrogen (pH), partial pressure of oxygen (pO_2_), total carbon dioxide (TCO_2_), bicarbonate (HCO_3_), base excess (BE), haematocrit (HCT). Gross laryngeal and laryngotracheal histopathology evaluation: Evidence of inflammation, haemorrhage and necrosis using scoring system of 1–4: 1 = minimal 2 = mild 3 = moderate 4 = severe. Distribution focal, multifocal, diffuse using scoring system of 1–3: 1 = focal 2 = multifocal 3 = diffuse. Statistically significant differences were determined as those with a P value of less than 0.05.
Main Findings (relevant to PICO question)	Placement time of devices: It took significantly longer to endoscopically intubate rabbits (median placement time 48 seconds, range 20–126 seconds) versus v-gel® placement (median placement time 6 seconds, range 2–20 seconds) (P = 0.003). Number of attempts required for placement of devices: All v-gel® placements required only one attempt; two endotracheal tube intubations required three attempts. There was no statistically significant difference (P = 0.17). Required level of isoflurane concentrations to maintain surgical plane of anaesthesia: There was no statistical difference (P = 0.59). Arterial blood gas values v-gel® had statistically significant (P = 0.045) increased levels of pCO_2_ with an increased mean of 7.5 mmHg (95% confidence intervals -0.9–14.1 mmHg) compared with endotracheal intubation (ETT) but the effect of time was not significant. All other values / differences were not statistically significant (P >0.05). Gross laryngeal and laryngotracheal histopathology evaluation: Airway trauma was present in histopathological examination of both groups but there was no statistically significant difference between groups (P >0.05). It should also be noted that when changing position of the subject, the v-gel® device was more easily displaced which although was not compared statistically is an important consideration.
Limitations:	All intubations and v-gel® placements were performed by the same experienced anaesthetist making is difficult to extrapolate to general practitioners who may have less experience. Using different operators would mean skill level would have varied, which may have affected the time to place devices. The anaesthetist was also not blinded and may have improved in skills over time with repeated procedures. The same breed and procedure may impact application to the general rabbit population and different surgical procedures. Intermittent positive pressure ventilation (IPPV) was not used in this study and thus airway sealing pressures were not assessed. The small sample size may reduce the power of the results. A type II error is possible as the sample size was limited for categorical data analysis.

**Toman et al. (2015):** 

Population:	Adult New Zealand White rabbits 2.5–3 kg, laboratory animals with no clinical abnormalities detected.
Sample size:	24 rabbits.
Intervention details:	Subjects were allocated to four groups with six animals in each group, and anaesthetised four times with the order of airway device being randomised: Endotracheal intubation (ETT). Laryngeal mask airway (LMA). Perilaryngeal airway (PLA). v-gel® rabbit (v-gel®). Blood pressure, heart rate and electrocardiogram (ECG) were measured at 1 minute, 5 minutes, and 30 minutes after placement of devices and establishment of anaesthesia. Xylazine and ketamine were used for premedication. Rocuronium bromide was used to suppress respiration –after complete paralysis the devices were placed. Animals were manually ventilated with 1.15% isoflurane with 50% air and oxygen mixture under 15 cm H_2_O pressure. Animals that regained spontaneous respiration after 30 minutes were given neostigmine and atropine prior to extubation.
Study design:	Randomised controlled trial.
Outcome studied:	Q-T intervals corrected for heart rate (QTc) intervals: QT duration determined measuring time from onset of Q wave to end of T wave with QT interval measured with Bazett formula. Mean arterial pressure (MAP): Invasive reading through left auricular artery. Blood gas values. Heart rate. All above parameters were measured at baseline, 1, 5, 15 and 30 minutes. Statistically significant differences were determined as those with a P value of less than 0.05.
Main Findings (relevant to PICO question)	QTc intervals at baseline, 1, 5, 15 and 30 minutes: There was no statistically significant difference between groups for baseline intervals. The QTc intervals at 1 and 5 minutes was significantly increased in the ETT group (approximately 285 milliseconds and 292 milliseconds respectively) compared to other groups which were all less than approximately 262 milliseconds (P <0.05). There was a significant increase (P <0.05) in QTc interval at 15 and 30 minutes between the PLA (approximately 255 milliseconds) and v-gel® groups (approximately 251 milliseconds). There was a significantly higher(P<0.05) QTc interval in the LMA group (approximately 260 milliseconds) at the 5^th^ minute compared to the v-gel® group (approximately 248 milliseconds). Mean arterial pressure (MAP): There was no statistically significant difference in all groups at baseline, 1, 5, 15 and 30 minutes. There was a significant difference in MAP value at 5 minutes when comparing the ETT (approximate MAP = 86) and v-gel® group (approximate MAP = 69). Blood gas values: There was no statistically significant difference between groups at baseline, 10 and 30 minutes**.** Heart rate: There was no statistically significant difference between the baseline for all groups. The heart rate values of the ETT group (approximately 180–205 bpm) were significantly higher than the PLA (approximately 160–170 bpm) and v-gel® (approximately 140–170 bpm) group (P <0.05). The heart rate in the LMA group (approximately 170–190 bpm) was significantly higher than the PLA (approximately 160–170 bpm) and v-gel® group (approximately 140–170 bpm) at 1, 5, 15 and 30^t^ minutes after intubation (P <0.05).
Limitations:	All intubations, v-gel® placements and manual ventilation was performed by the same anaesthetist making it difficult to extrapolate to general practitioners who may have less experience. Differences in operator skills may have changed which results were statistically significant in this study. The anaesthetist was also not blinded and may have improved in skills over time with repeated procedures. Exclusion of rabbits with ‘no clinical abnormalities’ lacks detail and specifics such as ASA status, age, health status which may have affected study outcomes. Small sample size may limit power of results. Same breed / weight and lack of surgical procedure may impact application to the general rabbit population and common surgical procedures under general anaesthesia. The results were provided in graphs with no numerical data or standard deviations given so it was difficult to extrapolate specific differences between factors such as heart rate, QTc interval and MAP.

**Wenger et al. (2017):** 

Population:	Adult female New Zealand White rabbits (7 months old), healthy based on physical examination. Rabbits showing signs of upper respiratory obstruction or oxygen saturation (SPO_2_) of less than 90% for more than 60 seconds were excluded.
Sample size:	Initial sample size: 10 rabbits. Final sample size: nine rabbits (reasons for exclusion addressed in section Intervention details).
Intervention details:	Subjects were acclimated to the environment for 1 week prior to experiment. Subjects were not fasted prior to experiments. Each subject was anaesthetised four times for each of the four-airway device. Subjects randomly allocated to four groups: Endotracheal tube (ETT) Laryngeal mask (LM). v-gel® supraglottic airway device (v-gel®). Face masks (FM). Subjects were sedated with fentanyl citrate and fluanisone. After 25 minutes the subjects were induced with propofol with an initial dose of 1 mg/kg. Jaw tone, palpebral reflex, reaction to lidocaine spraying of larynx and protraction of tongue were assessed to determine if the subject was suitably induced for placement of the airway devices. Propofol doses were repeated until successful placement of airway devices. The devices were placed blindly (without use of endoscope). The leakage of each device during spontaneous ventilation was assessed by calculation of difference between expiratory and inspiratory tidal volume over ten breaths. A computed tomography (CT) scan of the head, neck and abdomen was performed. Rocuronium bromide was administered and once apnoea was present, controlled mechanical ventilation (CMV) was started at 30 breaths per minute. Leakage was measured for increments of 2 cmH_2_O from 6 cmH_2_O to 15 cmH_2_ Exclusions: One rabbit had to be excluded from the CMV experiment due to signs of upper airway obstruction.
Study design:	Randomised crossover experimental trial.
Outcome studied:	Required dose of propofol. Time taken to secure airway device. Number of attempts to secure airway device. Airway leakage at different peak inspiratory pressures (PIP). CT of head, neck, abdomen to evaluate: Position of the SGAD and measurements of the larynx for the FM, v-gel® and LM groups. Severity of the laryngeal compression caused by the SGAD (measured in SGAD application only). Measure the total volume of stomach and gas present dorsally, a sign of gastric tympany due to controlled mechanical ventilation (CMV). Statistically significant differences were determined as those with a P value of less than 0.05.
Main Findings (relevant to PICO question)	Required dose of propofol: Significantly less propofol (P <0.05) was needed in the FM (2.0 mg/kg ± 0.5 mg/kg) group compared with ETT (5.5 mg/kg ± 1.4 mg/kg), LM (4.8 mg/kg ± 1.2 mg/kg) and v-gel® (5.1 mg/kg ± 2.1 mg/kg) groups. There was no significant difference between the other groups (P >0.05). Time taken to secure airway device: Significantly less time was needed in the FM group (82 seconds ± 34 seconds) compared with ETT (315 seconds ± 147 seconds), LM (275 seconds ± 89 seconds) and v-gel® (302 seconds ± 124 seconds) groups (P <0.001). There was no significant difference between the other groups (P >0.05). Number of attempts to secure airway device: Significantly less attempts were needed in the FM group (one attempt, range 1–1) compared with ETT (one attempt, range 1–10), LM (one attempt, range 1–2) and v-gel® (one attempt, range 1–4) groups (P <0.029). There was no significant difference between the other groups (P >0.05). Airway leakage at different peak inspiratory pressures (PIP): There was significantly more leakage at lower PIP in the FM group (PIP 6–8 centimetres of water [cmH_2_O] compared with the ETT (PIP > 16) , v-gel® (PIP 6 to >16 cmH_2_O) and LM (6 to >16 cmH_2_O) groups (P = 0.0001). The v-gel® (PIP 6 to >16 cmH_2_O) group showed a leak at significantly (P <0.05) lower pressure than ETT (PIP > 16). CT of head, neck, abdomen: The CT scans showed significantly smaller laryngeal widths (P = 0.004) and heights (P = 0.001) of the larynx in the v-gel® (width 1.9 mm ± 1.0 mm, height 2.8 mm ± 2.2 mm) group compared with the FM (width 2.3 mm ± 0.6 mm, height 5.8 mm ± 1.0 mm) and LM (width 3.1 mm ± 0.5 mm, height 6.2 mm ± 1.3 mm). The measurements were not done for the subjects in the ETT group. Severe laryngeal compression was seen in one v-gel® subject, with moderate laryngeal compression being seen in two v-gel® subjects although significant differences between groups was not assessed. There was a significant increase in gas in the stomach in the FM (0.2 cm^3^, -1.6–1.5) and LM (0.1 cm^3^, -1.0–^-^1 cm^3^) groups (P = 0.007) compared to ETT (-0.3 cm^3^, -1.1–6 cm^3^ and v-gel® (-0.5 cm^3^, -3.9–0.0 cm^3^). There was no significant difference in total gastric volume between groups before and after CMV (P >0.05).
Limitations:	Airway devices were placed by board certified anaesthetists so results may not be extrapolatable to general practitioners. Small sample size may limit power of results. Same breed / weight and lack of surgical procedure may impact application to the general rabbit population. The four treatments were applied to the same animal and this could have lead to laryngeal trauma that made application of a later device more difficult. The degree of tympanism could have been more profound as the gas was not assessed within the ingesta of the stomach, only in the dorsal gaseous phase. For CT scans the ETT group did not have width and height measurements so it is not possible to compare possible differences with other groups. The authors commented on laryngeal compression noted in CT scans for three v-gel® subjects but did not give us a comparison to other groups so it is not possible to determine the significance of this.

**Cruz et al. (2000):** 

Population:	Female Norfolk rabbits (2.4–3.1 kg).
Sample size:	Eight rabbits.
Intervention details:	Subjects were anaesthetised twice 1 week apart. All eight subjects were intubated with endotracheal tube during first anaesthesia. All eight subjects had laryngeal masks applied during second anaesthesia. Subjects were premedicated with methotrimeprazine, thiopentone was used for induction intravenously. 5 ml of barium sulphate as a radiographic contrast was administered into the stomach via an intragastric tube. Intubation was attempted by the same inexperienced operator for two attempts. Following this, an experienced operator performed intubation. A Bain circuit was used to maintain anaesthesia for 60 minutes with isoflurane. Values were recorded for heart rate, blood pressure (direct via auricular artery), electrocardiogram (ECG), arterial blood gases, oxygen saturation, tidal volume, temperature and respiratory rate every 15 minutes. Immediately after removal of the endotracheal tube or laryngeal mask, thoracic and cervical radiographs were taken to assess for regurgitation and aspiration of the contrast.
Study design:	Repeated measure trial (conference proceeding).
Outcome studied:	Regurgitation of stomach contents following anaesthesia assessed via radiographs. The dose of thiopentone required. The difference between anaesthetic parameters: Heart rate, blood pressure, ECG, arterial blood gas, oxygen saturation, tidal volume, temperature and respiratory rate. The number of attempts taken to apply the airway device. Statistically significant differences were determined as those with a P value of less than 0.05.
Main Findings (relevant to PICO question)	Regurgitation of stomach contents following anaesthesia assessed via radiographs: There was no significant regurgitation in either group (P >0.05). The dose of thiopentone required: A significantly (P <0.05) higher dose of thiopentone was required for endotracheal intubation (36 mg/kg) compared to laryngeal mask application (24 mg/kg). The difference between anaesthetic parameters: There was no significant difference between the groups for any variable (P >0.05). The number of attempts taken to apply the airway device: Endotracheal intubation was successful in 3/8 animals at the first attempt. Laryngeal masks were placed in all eight animals at the first attempt but there was no statistically significant difference between groups (P >0.05).
Limitations:	Small sample size may limit power of results. Lack of surgical procedure may impact application to the general rabbit population. Rabbits were larger breeds weighing between 2.3 and 3.1 kg which limits application to smaller dwarf breeds commonly seen in clinical practice. There were no details on exclusions / inclusions for subjects and as such we do not know if age / health status impacted results. ‘Norfolk’ is not a recognised rabbit breed so it is unclear which breed of rabbit was used. The same inexperienced practitioner performed all airway device applications but only had two attempts before an experienced practitioner stepped in. In clinical practice more than two attempts may be required which makes it difficult to extrapolate the results to general practitioners. The inexperienced practitioner may also have improved in technique as the study progressed. Confidence intervals and ranges were not reported so it is more difficult to interpret statistically significant results.

## Appraisal, application and reflection

Facemasks are commonly used in first opinion practice for rabbit patients due to the difficulty of endotracheal intubation by veterinarians with limited experience with rabbit patients. Current evidence suggests that face masks are inadequate for airway maintenance as it is difficult to achieve an effective seal to provide adequate oxygen leading to hypoxaemia, hypercapnia and an inability to achieve positive pressure ventilation in the event of apnoea (Wenger et al., 2017). Endotracheal tubes have been used successfully in animal practice for many years and when using a technique allowing visualisation of the trachea, are an excellent choice for maintaining a patent airway in rabbit patients. The use of the blind technique can result in mucosal damage and multiple intubation attempts. For those practitioners who are untrained in endotracheal intubation in rabbits, supraglottic devices may be an alternative. There are a range of supraglottic devices available including the rabbit specific v-gel®. Both endotracheal tubes and supraglottic airway devices have evidence demonstrating their effective use in maintaining anaesthesia with no significant differences in anaesthetic parameters.

All studies used subjects that were healthy and young and with low American Society of Anesthesiologists (ASA) scores and only one study assessed the use of both devices whilst the subjects were undergoing a procedure (Comolli et al., 2020). Only one study assessed the recovery of patients (Crotaz, 2013), and the subjects of two studies were euthanised whilst still under anaesthesia (Engbers et al., 2017; and Comolli et al., 2020). Considering this, it is difficult to extrapolate the findings to rabbits seen in general practice who would be put under anaesthesia for surgical procedures where recovery is an important part of an anaesthetic protocol. Rabbit patients may also present with higher ASA scores due to age or disease and selection of premedication and induction agents may be limited by clinic protocol, availability of different agents and patient requirements. Additionally, no studies looked at dwarf breeds and whether application of a supraglottic device or endotracheal tube was easier or possible given the smaller oral cavity size. Majority of studies used New Zealand White rabbits as subjects (Enbers et al., 2017, Comolli et al., 2020, Tman et al., 2015; and Wenger et al., 2017). This breed is commonly used for research purposes and as such the results of these studies may be more readily applicable in a research setting.

Crotaz et al. (2010) concluded that the v-gel® supraglottic airway device was simpler to use which is further supported by other studies which demonstrated that the time to place v-gel® was faster, more consistent and required fewer attempts when compared to endotracheal intubation (Crotaz, 2010; Cruz et al., 2000; Engbers et al., 2017; and Comolli et al., 2020). As induced patients are at risk of apnoea, the length of time it takes to achieve a patent airway is critical to prevent hypoxaemia (Navarrete-Calvo et al., 2014). This is even more clinically significant when considering patients in respiratory arrest where fast airway access is imperative. Cruz et al. (2000) demonstrated that a significantly higher dose of induction agent, in this case thiopentone, was required to achieve endotracheal intubation than supraglottic device insertion^15^. However, the studies premedicated and induced their patients with a variety of different agents, including induction chamber or facemask with isoflurane, intravenous administration of thiopentone, propofol, ketamine and xylazine or alfaxalone. The use of different agents could impact the ease or difficulty of the application of different airway devices as would impact sedation quality at induction and the stress levels of each subject. In patients where using less anaesthetic agents would be ideal such as geriatric patients or patients with organ dysfunction, supraglottic devices may be a the better choice if available premedication and inductions drugs at higher dosages increases risk for these patients.

Power calculations were only undertaken in one study (Wenger et al., 2017). As all studies had small sample sizes, it is possible that differences between the groups were only insignificant because there were not enough subjects in each group, resulting in a type 2 error. When comparing mucosal damage to the trachea, Engber et al. (2017) found histological evidence of damage to the tracheal mucosa was significantly higher when using an endotracheal tube, whereas Comolli et al. (2020) found no significant difference between either device (Engbers et al., 2017; and Comolli et al., 2020). Whilst this could be a type 2 error due to small sample size, it could also be explained by the fact that the Engber et al. (2017) study used the blind technique, resulting in multiple intubation attempts and leading to more mucosal damage. There could also be bias in histological scoring. Comolli et al. (2020) in contrast visualised the trachea during endotracheal intubation, thus reducing the attempts required to achieve intubation. The Wenger et al. (2017) study showed that more attempts were required to place the endotracheal tube than there were with the supraglottic device, which could explain why there was further damage to the oral cavity and tracheal mucosa. Use of the blind technique could also increase the risk of oesophageal perforation (Ranchère et al., 1992) and this must also be considered as it may have long term effects on patients. Long-term recovery was not assessed in these studies; thus, it is not possible to assess whether mucosal damage to the trachea has significance to a patient’s recovery and future health or is clinically significant. Other studies have shown that tracheal damage can lead to tracheal strictures which can increase patient morbidity and mortality (Grint et al., 2006).

Bateman et al. (2005) provided good evidence that supraglottic devices could be used successfully in rabbit patients but resulted in gastric tympanism in some cases when positive pressure ventilation was provided. Further investigation is warranted to see if gastric tympany has any long-term effects of clinical significance, especially when considering that gut stasis is a common complication of recovery. Cruz et al. (2000) by use of barium sulphate as a radiographic contrast directly into the stomach of the subjects concluded that there was no regurgitation present on radiographs following use of endotracheal tubes or supraglottic devices. However, one should note that gastric oesophageal reflux is highly unlikely in rabbits due to their strong cardia preventing backflow of gastric contents. Crotaz et al. (2010) and Bateman et al. (2005) noted that there was no evidence that the supraglottic v-gel® device would protect against aspiration of fluids and was displaced easily during patient movement, increasing the likelihood of aspiration (Bateman et al., 2005; Crotaz, 2013; and Comolli et al., 2020). Dental procedures are common in rabbit patients, and it may be risky to use supraglottic devices without knowing if the seal is adequate to prevent aspiration considering the fluid produced and introduced into the patient’s oral cavity via dental instruments.

Engbers et al. (2017) used computed tomography (CT) scans to measure the height and width of the larynx, showing that it was significantly narrower in subjects using the v-gel® supraglottic device when compared to the laryngeal mask airway device or facemask. The Wenger et al. (2017) study used CT scans to assess the correct placement of the v-gel® supraglottic device where half of the subjects required readjustment with three subjects showing laryngeal compression with increased mucous accumulation. This is an important consideration when using supraglottic devices as narrowing of the larynx due to the device size or mucous accumulation can lead to laryngeal compression which may result in an increased breathing effort as a result of increased airway resistance. Considering this, monitoring of respiratory effort when using supraglottic devices is warranted, although the clinical significance of increased breathing effort was not discussed.

Whilst a device may be effective for spontaneous ventilation, positive pressure ventilation or controlled mechanical ventilation may be required in the event of apnoea. Bateman et al. (2005) showed that in four of the six subjects using supraglottic devices, gastric tympany was noted and there was leakage of anaesthetic gas. This suggests whilst controlled mechanical ventilation can be achieved with supraglottic devices, there is leakage into the environment and the alimentary tract, which can make it more difficult to provide adequate ventilation in apnoeic patients. This would also potentiate environmental contamination with the gaseous anaesthetic agent which is an important consideration for the safety of staff and other patients. Bateman et al. (2005) suggested that keeping the pressure below 14 cmH_2_O could mitigate some leakage however, this would likely also decrease alveolar ventilation and lead to hypercapnia. Comolli et al. (2020) demonstrated that endotracheal intubation is the most effective device to decrease dead space as supraglottic devices showed higher levels of PaCO2. However, in the event of unsuccessful endotracheal intubation, supraglottic devices are still superior to facemasks as controlled mechanical ventilation has been shown to lead to much more significant leakage, increased dead space and PaCO_2_ in facemasks compared to supraglottic devices (Bateman et al., 2005; and Wenger et al., 2017).

More research is warranted in clinic specific settings to determine the suitability of supraglottic airway devices in rabbit patients. The easier loss of airway seal during movement when using a supraglottic device is not ideal in procedures that require repositioning. They also take up more space in the oral cavity, making them difficult to use in dental procedures, and potentially more difficult to use in smaller breeds. v-gels® do have the benefit of being easily sterilised via autoclave which can reduce bacterial growth and contamination which can be important in patients where respiratory defences are compromised. They may also be easier to apply in rabbit patients, providing an alternative for the practitioner lacking the experience or equipment to perform endotracheal intubation safely. Endotracheal intubation is more effective than supraglottic devices in achieving positive pressure ventilation in the case of apnoea and may be a better option for dental procedures as less space is taken up in the oropharynx and the better seal reduces the risk of aspiration. It should be noted however, that the blind technique has a higher risk for mucosal damage and increased likelihood of repeated intubation attempts.

The current evidence demonstrates that both devices can be used effectively to maintain anaesthesia in rabbit patients and that the final choice of airway maintenance device should be based on availability of equipment, the training of the practitioner and the procedure to be undertaken.

## Methodology Section

**Search Strategy:** 

Databases searched and dates covered:	CAB Abstracts via Web of Science Platform (1973–present) Medline via OVID SP (1946–present)
Search strategy:	CAB Abstracts: TI=(rabbit OR rabbits) AND TI=(intubation OR supraglottic OR endotracheal v-gel OR airway) AND TS=(anaesthetic OR anaesthesia OR anesthetic OR anesthesia) AND LA=(English) Refined by: RESEARCH AREAS: (VETERINARY SCIENCES) Timespan: All years. Indexes: CAB Abstracts. Medline: ((rabbit or rabbits) and (intubation or supraglottic or endotracheal v-gel or airway) and (anaesthetic or anaesthesia or anesthetic or anesthesia)).m_titl. limit 1 to English language
Dates searches performed:	30 Dec 2021

**Exclusion / Inclusion Criteria:** 

Exclusion:	Articles not relevant to the PICO question (not direct comparison of endotracheal intubation and supraglottic devices), article summaries, literature reviews, case reports (provided no comparison as were reports on individual patients), case series (comparison over time not relevant), non-peer reviewed conference proceedings, articles not in English language.
Inclusion:	Articles relevant to the PICO question (comparisons of endotracheal intubation and supraglottic devices), randomised controlled trials, cohort studies, case control studies, cross-sectional studies, peer-reviewed conference proceedings.

**Search Outcome:** 

Database	Number of results	Excluded – Case study / report	Excluded – Summary article	Excluded – Not relevant to PICO question	Total relevant papers
CAB Abstracts	36	1	8	22	5
Medline	11	1	0	6	4
Total relevant papers when duplicates removed					5

## Conflict of interest

The authors declare no conflicts of interest.
